# Serum metabolomics in early pregnancy differentiates between gestational hypertension and preeclampsia

**DOI:** 10.1186/s12884-026-09168-0

**Published:** 2026-04-29

**Authors:** Tahmineh R. Kalbasi, Carla Janzen, Yang Cheng Hu, Mary M. Nellis, David Elashoff, Sherin U. Devaskar

**Affiliations:** 1 The Department of Medicine Statistics Core, David Geffen School of Medicine, University of California, Los Angeles, CA USA; 2 Department of Obstetrics and Gynecology, Division of Maternal Fetal Medicine, David Geffen School of Medicine, University of California, Los Angeles, CA USA; 3 Fielding School of Public Health, University of California, Los Angeles, CA USA; 4 Clinical Biomarkers Laboratory, Department of Medicine, Emory University, Atlanta, GA USA; 5 Department of Pediatrics, David Geffen School of Medicine, University of California, Los Angeles, CA USA

**Keywords:** High-resolution metabolomics, Pregnancy outcomes, The PARENTs cohort, Gestational hypertension, Preeclampsia, Hypertensive disorders of pregnancy

## Abstract

**Background:**

Early identification of pregnancies at risk for gestational hypertension (gHTN) and preeclampsia (PE) remains a major clinical challenge. We investigated whether early‑pregnancy serum metabolomic profiles differentiate gHTN and PE prior to clinical onset.

**Methods:**

High-resolution metabolomics (HRM) analysis was performed on 126 early-pregnancy serum samples collected at ≤ 20 weeks’ gestation from 97 pregnant women enrolled in the Placental Assessment in Response to Environmental Pollution study (PARENTs) cohort at UCLA. Metabolic profiles were compared among pregnancies that later developed gestational hypertension (gHTN; *n* = 14 mothers, 20 samples), preeclampsia (PE; *n* = 9 mothers, 11 samples), and pregnancies without ischemic placental disease (non-IPD; *n* = 74 mothers, 95 samples). Untargeted metabolome-wide association studies (MWAS) and pathway enrichment analyses were conducted. Multivariable linear regression models adjusted for key maternal and pregnancy characteristics such as maternal age, race/ethnicity, early-pregnancy BMI, fetal sex, and parity were used to evaluate associations.

**Results:**

Distinct metabolic profiles differentiated gHTN and PE from non-IPD pregnancies. Alterations in the tryptophan metabolism pathway were observed in both gHTN and PE, with a significant dose–response relationship across groups (non-IPD > gHTN > PE, *p* = 0.008). Key metabolites, including tryptophan and its derivatives, were progressively depleted in association with increasing disease severity. Additionally, urea cycle metabolism was altered in gHTN, with higher levels of arginine and citrulline linked to nitric oxide production and vascular tone regulation. Comparisons between PE and gHTN revealed additional differences, including lower concentration of phenylalanine and pantothenic acid in PE, suggesting distinct metabolic alteration.

**Conclusion:**

Early‑pregnancy metabolomic signatures reveal both shared and condition‑specific metabolic pathways underlying gHTN and PE, with tryptophan metabolism showing a dose–response relationship indicative of disease severity. These early gestational alterations may serve as biomarkers for hypertensive disorders of pregnancy (HDP), enabling closer monitoring and stratification in high-risk pregnancies. Further studies in larger cohorts are needed to validate these findings and explore therapeutic implications.

**Supplementary Information:**

The online version contains supplementary material available at 10.1186/s12884-026-09168-0.

## Background

Hypertensive disorders of pregnancy (HDP) collectively contribute to considerable morbidity and mortality in mothers and offspring worldwide and impact 13–15% of U.S. pregnancies and account for 16% of maternal deaths in well-resourced countries [1–3]. Preeclampsia (PE), defined by hypertension and proteinuria after the 20th week of gestation, affects approximately 3–8% of pregnancies in the United States. As these conditions pose considerable risks to both maternal and fetal health, we aim to identify early-pregnancy metabolic signatures characteristic of PE and gestational hypertension (gHTN), with a particular focus on distinguishing features prior to 20 weeks of gestation. Although PE and gHTN share clinical features, studies indicate they exhibit distinct metabolomic profiles before clinical onset, supporting separate expletory analyses to uncover early patterns that may lead to each condition [4–6]. Metabolic alterations and patterns associated with these conditions may enhance our understanding of underlying mechanisms that might become treatable now or in the future.

High-throughput metabolomics technology offers a new tool that can help us improve our understanding of the metabolic mechanisms of diseases in humans. High-resolution metabolomics based on liquid chromatography coupled with high-resolution mass spectrometry (LC-HRMS) and sophisticated data extraction algorithms facilitates the analysis of a wide array of molecules in biological samples. This approach gave credence to the emergent concept of liquid biopsy. Untargeted approaches generate comprehensive profiles of small molecules (metabolites) that may also be associated with a particular disease or diagnosis [7]. Most importantly, such investigations have suggested novel pathways and generated new hypotheses beyond conventionally accepted ones currently in play. In recent years, the number of studies that have employed HRM has grown; however, very few have focused on metabolic pathways perturbed early in pregnancy that may be useful in helping to anticipate the subsequent development of adverse pregnancy outcomes [8–11]. To date, there are only two untargeted maternal serum metabolomic studies involving both PE and gHTN [5, 6]. In the present study, we generated metabolomic profiles from serum samples collected between 10 and 20 weeks of gestation from pregnant women enrolled in the prospective cohort of the ‘Imaging Innovations for Placental Assessment in Response to Environmental Pollution study (PARENTs) at the University of California, Los Angeles (UCLA) [12]. This cohort consists of high-risk and racially diverse pregnant women living in Los Angeles County.

## Material and methods

### Study design and population

The PARENTs cohort is a prospective cohort (2016–2019) that followed 199 consented pregnant women from early pregnancy upon recruitment from antenatal clinics at the University of California, Los Angeles [12]. Women were eligible to be enrolled in the study if they had a viable singleton gestation early in pregnancy. Exclusion criteria were maternal age < 18 years, fetal malformation evident before or after enrollment, known fetal chromosomal abnormalities, twin pregnancy, plans to terminate the pregnancy, contraindications for obtaining a magnetic resonance imaging (MRI) in pregnancy, or inability to provide consent [12]. The present metabolomics analysis represents a biospecimen‑based subset of the PARENTs cohort for which early‑pregnancy serum samples were available and analyzed through the NIH Children’s Health Exposure Analysis Resource (CHEAR) program [13]. Participants provided informed consent and completed up to three visits during pregnancy, at which clinical characteristics and biospecimens were collected [12]. Gestational age was determined by first‑trimester ultrasound. The study protocol was approved by the University of California, Los Angeles (UCLA) Institutional Review Board and conducted in accordance with the Declaration of Helsinki. Study details are available at ClinicalTrials.gov (Identifier: NCT02786420, registered May 25, 2016).

### Analytic cohort definition

**Fig. 1:**
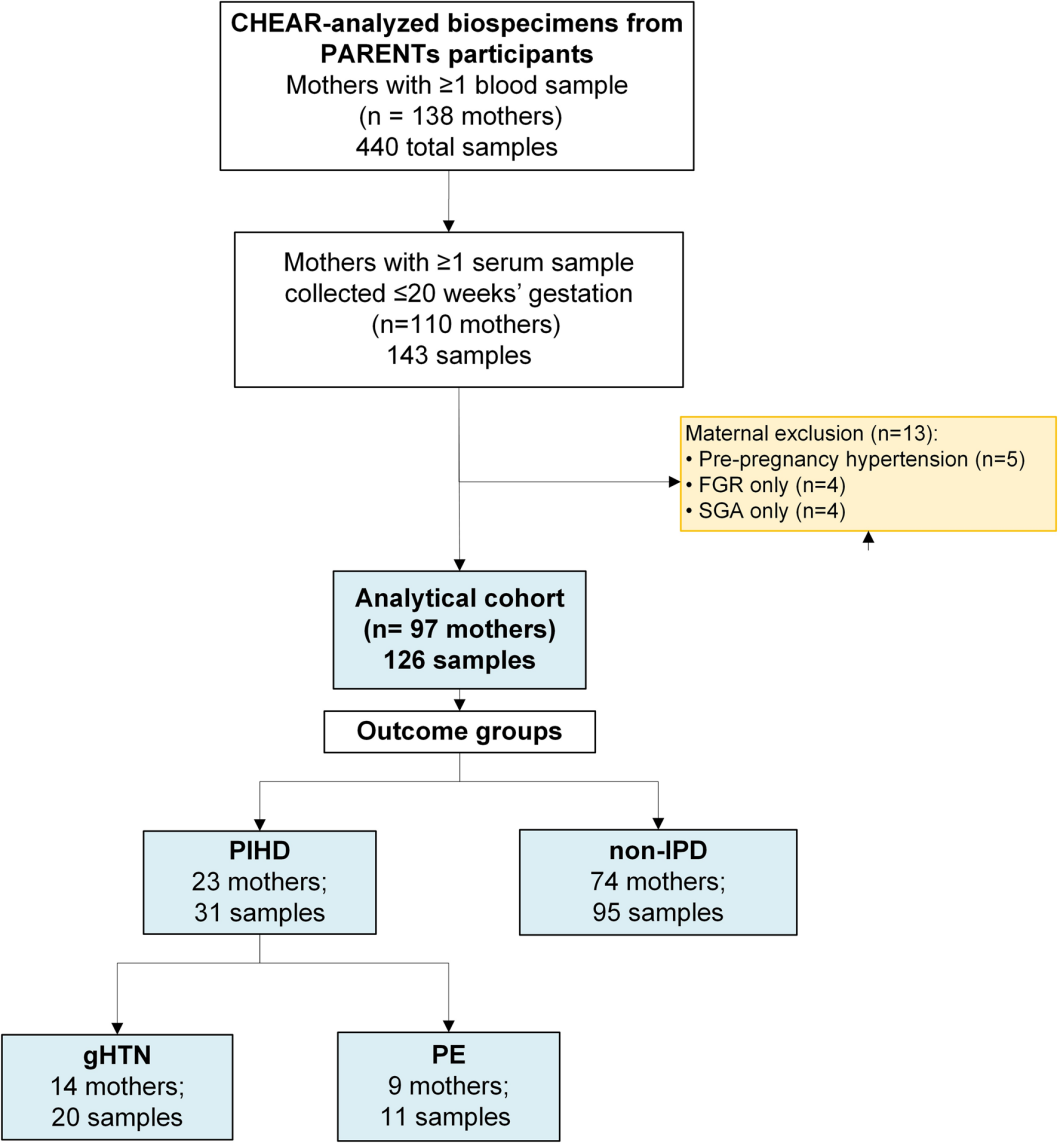
Selection of the early‑pregnancy metabolomics analytic cohort

Among the 199 women enrolled in the PARENTs cohort, 110 contributed at least one serum sample collected at ≤ 20 weeks’ gestational age, yielding 143 early‑pregnancy serum samples. In this study, we focused on hypertensive disease arising during pregnancy (gHTN or PE). Women with pre‑existing chronic hypertension were excluded a priori to isolate pregnancy‑onset hypertensive disorders; accordingly, the comparison group represents pregnancies without IPD (non‑IPD). For the present analysis, we therefore excluded women with pre‑existing chronic hypertension (*n* = 5), isolated fetal growth restriction (FGR; *n* = 4), or small‑for‑gestational‑age (SGA) without hypertensive disease (*n* = 4). The final analytic sample consisted of 97 mothers contributing 126 serum samples collected ≤ 20 weeks’ gestation (Fig. 1).

### Unit of analysis

Maternal demographic and clinical characteristics are reported at the individual level (*n* = 97 mothers; Table 1). High‑resolution metabolomics analyses were conducted at the sample level, as some participants contributed more than one early‑pregnancy serum sample prior to disease onset. Hypertensive disorder classification (non‑IPD, gHTN, or PE) was assigned at the maternal level based on pregnancy outcome, and all analyzed samples were collected prior to clinical onset of disease. A total of 126 early-pregnancy serum samples were included in metabolomics analyses, including 95 from non-IPD pregnancies, 20 from pregnancies that developed gHTN, and 11 from pregnancies that developed PE.

**Table 1:** Maternal descriptive statistics stratified by non-IPD and PE and gHTN

Variable	non-IPD, *n* = 74	PE, *n* = 9	gHTN, *n* = 14	*P* value
Maternal age (years), median(IQRs)	33 (31–35)	33 (32–34)	34 (30–37)	0.7
Race and ethnicity, n(%)				0.5
White, non-Hispanic	33 (44.6)	3 (33.3)	9 (64.3)	
Hispanic	13 (17.6)	3 (33.3)	1 (7.1)	
Asian	22 (29.7)	3 (33.3)	2 (14.3)	
Black or AA	6 (8.1)	0 (0)	2 (14.3)	
Mother’s Education, n(%)				0.9
Bachelor or higher education	65 (87.8)	9 (100)	13 (92.9)	
High school graduate or some	9 (12.2)	0 (0)	1 (7.1)	
Nuliparity				0.4
No	40 (54.1)	4 (44.4)	5 (35.7)	
Yes	34 (45.9)	5 (55.6)	9 (64.3)	
Early-pregnancy BMI	22.5 (21.3–26.2)	22.3 (20.6–34.5)	23.5 (22.3–27.9)	0.2
Low-dose aspirin use, (n%)				0.07
No	70 (95.9)	8 (88.9)	11 (78.6)	
Yes	3 (4.1)	1 (11.1)	3 (21.4)	
Smoking status, n(%)				0.5
former smoker	14 (20.9)	0 (0)	1 (10)	
never smoker	53 (79.1)	8 (100)	9 (90)	
Gestational Diabetes Miletus, n(%)				0.7
No	66 (90.4)	8 (88.9)	12 (85.7)	
Yes	7 (9.6)	1 (11.1)	2 (14.3)	

Characteristics are reported at the maternal level (total *n* = 97 mothers)

^*^P-values for categorical variables were calculated using Fisher's exact test, and *P* values for continuous variables were calculated using the Kruskal Wallis test

### Outcome definitions

We considered ischemic placental disease (IPD) as a collective of conditions characterized by reduced blood flow to the placenta and uteroplacental ischemia. These include chronic hypertension (defined as a blood pressure (BP) reading of 140/90 mmHg or higher that either existed before pregnancy or appeared before the 20th week of gestation), gHTN, PE, fetal growth restriction, and SGA [12, 14, 15].

PE was defined as a BP reading of 140/90 mmHg or more on two separate instances spaced at least four hours apart after the 20th week of gestation in those subjects with a prior normal BP reading, coupled with proteinuria exceeding 300 mg/24 h. When proteinuria was absent, PE was characterized by new-onset of hypertension combined with the emergence of thrombocytopenia, compromised kidney function, liver function aberrations, pulmonary edema, or neurological or visual disturbances [12]. gHTN was diagnosed after 20 weeks of gestation without any signs of systemic involvement characteristic of PE [12]. The diagnosis of gHTN or PE was collectively defined as the pregnancy-induced hypertensive disorder (PIHD). The first sample was collected in the first or early second trimester, with a median gestational age of 13.8 weeks [interquartile range (IQR): 12.4–15.5 weeks].

### High-resolution serum metabolomics

HRM was conducted using LC-HRMS [16–18]. Serum samples were prepared and analyzed in batches of 40, each including pooled reference plasma and NIST SRM-1950 samples for quality control. After storage at −80 °C, samples were thawed on ice, vortexed, and 50μL aliquots were treated with 100μL of acetonitrile containing eight stable isotopic standards. After 30 min on ice and centrifugation, the supernatant was analyzed using HILIC chromatography with a Q Exactive HF mass spectrometer, operating in positive ion mode at 120,000 resolution and mass-to-charge ratio (*m/z)* 85–1270. Data were processed and aligned using apLCMS and xMSanalyzer, with batch correction by ComBat [19]. This analysis utilizes data specifically from the HILIC column operating in positive ion mode**.**

## Statistical analyses

### Descriptive analyses

We summarized continuous variables, such as maternal age and early-pregnancy body mass index (BMI), using medians and IQRs. Categorical variables, such as mother’s race and ethnicity, education, smoking status, and primigravida, were summarized using counts and percentages. To compare these characteristics across PE, gHTN, and non-IPD pregnancies, we used Fisher’s exact test for categorical variables and the non-parametric Kruskal–Wallis test for continuous variables. Descriptive analyses were conducted at the maternal level.

### Metabolomic data preprocessing and annotation

Only annotated metabolites and *m/z* features detected in at least 70% of the samples are included in the analyses; 6,644 metabolomic features were retained for analysis. In this context, non-detected values reflect peaks below the limit of detection rather than true missingness. For each metabolite meeting the detection threshold, non-detected values were imputed using the minimum observed concentration divided by two [20]. The data were then log2-transformed and normalized using a median centering approach [21, 22].

Metabolites were annotated using two approaches. Features were matched to an in-house reference library with a 10-ppm mass tolerance. In parallel, xMSannotator was applied to the full feature table using a 5-ppm mass tolerance, 10-s retention time (rt) window, Pearson correlation threshold of 0.6, maximum isotope search of five, and mass defect window of 0.01 to generate HMDB-based candidates. Significant features were then linked to annotation candidates using find Overlapping *m/z*s function in xMSanalyzer. Features were considered annotated if supported by the in-house library or by XMSannotator at a confidence level of three with biological relevance to the study context.

### Metabolome-wide association analyses

To perform untargeted metabolome-wide association analyses (MWAS), we constructed multivariable linear regression models to compare *m/z* features between pairs of groups (gHTN vs. non-IPD, PE vs. non-IPD, and PE vs. gHTN). Analyses were conducted at the sample level. Model coefficients were interpreted as the average log_2_ fold change (FC) in feature concentrations between groups. Models were adjusted for mother’s age, race, and ethnicity, early-pregnancy BMI (primarily measured from up to 6 months prior to pregnancy through < 14 weeks’ gestation), fetal sex, and primigravida (yes/no).

Missing early BMI values (*n* = 2) were imputed using maternal BMI measured at the first prenatal visit (< 13 weeks for both), a period during which maternal weight change is minimal. Low-dose aspirin use was defined using first-visit interview data. Interview data were cross-validated with hospital chart abstraction to confirm aspirin exposure prior to 20 weeks’ gestation, showing high concordance. When a mother had two samples collected before 20 weeks, each sample was treated as an individual data point in the analysis. A sensitivity analysis was conducted using robust standard errors to account for the effect of repeated metabolite data from the small number of mothers contributing more than one sample.

### Multiple testing and statistical significance

Given the small sample size and exploratory nature of this analysis, we used raw *p*-values for all visualizations. We also employed a Bayesian approach to account for multiple hypothesis testing. assuming a prior probability of 0.05 that a given metabolic feature is truly associated with the outcome, the corresponding raw *p*-value cutoffs yielding false discovery rate (FDR) of 0.1, 0.2, and 0.05 were 0.0105, 0.0047, and 0.0022, respectively [23]. The *m/z* features with raw *p*-values < 0.0022 were considered statistically significant (FDR < 0.05).

### Multivariate and pathway analyses

Partial least squares discriminant analysis (PLS-DA) identifies latent variables that best separate groups based on correlated metabolomic features, enabling assessment of group separation and identification of key metabolites using variable importance for projection (VIP) scores. We employed PLS-DA using the “ropls” package in R.

Features with a raw *p*-value < 0.05 and VIP > 1.5 were selected to perform pathway enrichment analysis using Mummichog v2.0 [24]. Pathway enrichment analysis in Mummichog v2 uses Fisher’s exact test to identify metabolic pathways that are overrepresented in the set of significant *m/z* features. This approach accounts for the complexity of metabolite mapping to a specific pathway by using a permutation procedure compute adjusted empirical *p*-values [24].

If the first latent component of the PLS-DA model did not demonstrate statistically significant group separation, pathway enrichment analysis was conducted using features meeting the Bayesian FDR-derived raw *p*-value threshold of 0.0105. Pathways were considered supported when at least four empirical compounds contributed to enrichment and at least one metabolite within the pathway was confirmed through annotation among overlapping empirical features. Annotation was used solely for post hoc pathway confirmation.

For *m/z* features corresponding to in-house confirmed metabolites, additional evidence was evaluated using Pearson correlation and visual inspection of scatter plots. When Mummichog suggested a best guess corresponding to a metabolite present in our in-house library but with differences in *m/z* or rt, concordance with the confirmed metabolite was assessed through correlation analysis. Furthermore, annotations were accepted for pathway confirmation when both Mummichog and XMSannotator independently assigned the same metabolite to an *m/z* feature.

### Data visualization and trend analyses

Type 1 and Type 2 Manhattan plots were utilized to display patterns of differential concentrations according to molecular mass and rt. The score plot from PLS-DA analyses was used to visualize participants’ classification according to metabolites discriminating between non-IPD, gHTN, and PE pregnancies. A volcano plot presents the log_2_ FC against -log_10_ (*p*-value) from multivariate regression models. To evaluate linear trends in metabolite concentrations across non-IPD, gHTN, and PE, we fit multivariable linear regression models including complication status (non-IPD, gHTN and PE) as an ordinal variable coded 0, 1, and 2, adjusting for age, race, fetal sex, early-pregnancy BMI, and nulliparity. The *p*-value associated with the ordinal complication variable in the multivariable linear regression model was used to assess the statistical significance of the linear trend. All tests were two-sided, and all analyses were performed using the R package, version 4.3.2 (2023–10–31).

## Results

### Study sample characteristics

Throughout the study, maternal characteristics and outcomes are reported at the mother level (*n* = 97), metabolomics analyses are performed at the sample level (*n* = 126 serum samples), and metabolomic measurements represent individual features (*n* = 6,644 m/z features). The median age of participants was 33 years (IQR 31 to 35 years). Racial composition was as follows: 45 mothers (46%) were non-Hispanic White, 17 (18%) were Hispanic, 27 (28%) were Asian, and 8 (8%) were of Black or African American ancestry. Most participants, 87 mothers (90%), had at least a bachelor's degree. Approximately half (*n* = 49; 51%) were primigravidae; among these, the majority were pregnant with their second child (37 of 49, or 76%). Most participants reported never smoking (*n* = 70; 72%), while 15% were former smokers (*n* = 15). Median early-pregnancy BMI was 22.6 kg/m^2^ (IQR 21.3–26.8 kg/m^2^), with no statistical differences observed in baseline characteristics between the clinical outcome groups. Low-dose aspirin use was infrequent overall (*n* = 7) and did not differ significantly between groups (*p* = 0.074). (Table 1).

Among the 97 pregnant women in the analytic cohort (126 total samples), 23 mothers (24%) developed PIHD and contributed 31 serum samples (20 samples from pregnancies that developed gHTN and 11 from pregnancies that developed PE), whereas 74 mothers (76%) without IPD contributed 95 samples. PIHD cases included PE (*n* = 9) and gHTN (*n* = 14).

Following preprocessing and quality control, 6,644 metabolomic features were retained for metabolomic analyses.

### Gestational hypertension vs. non-IPD

**Fig. 2:**
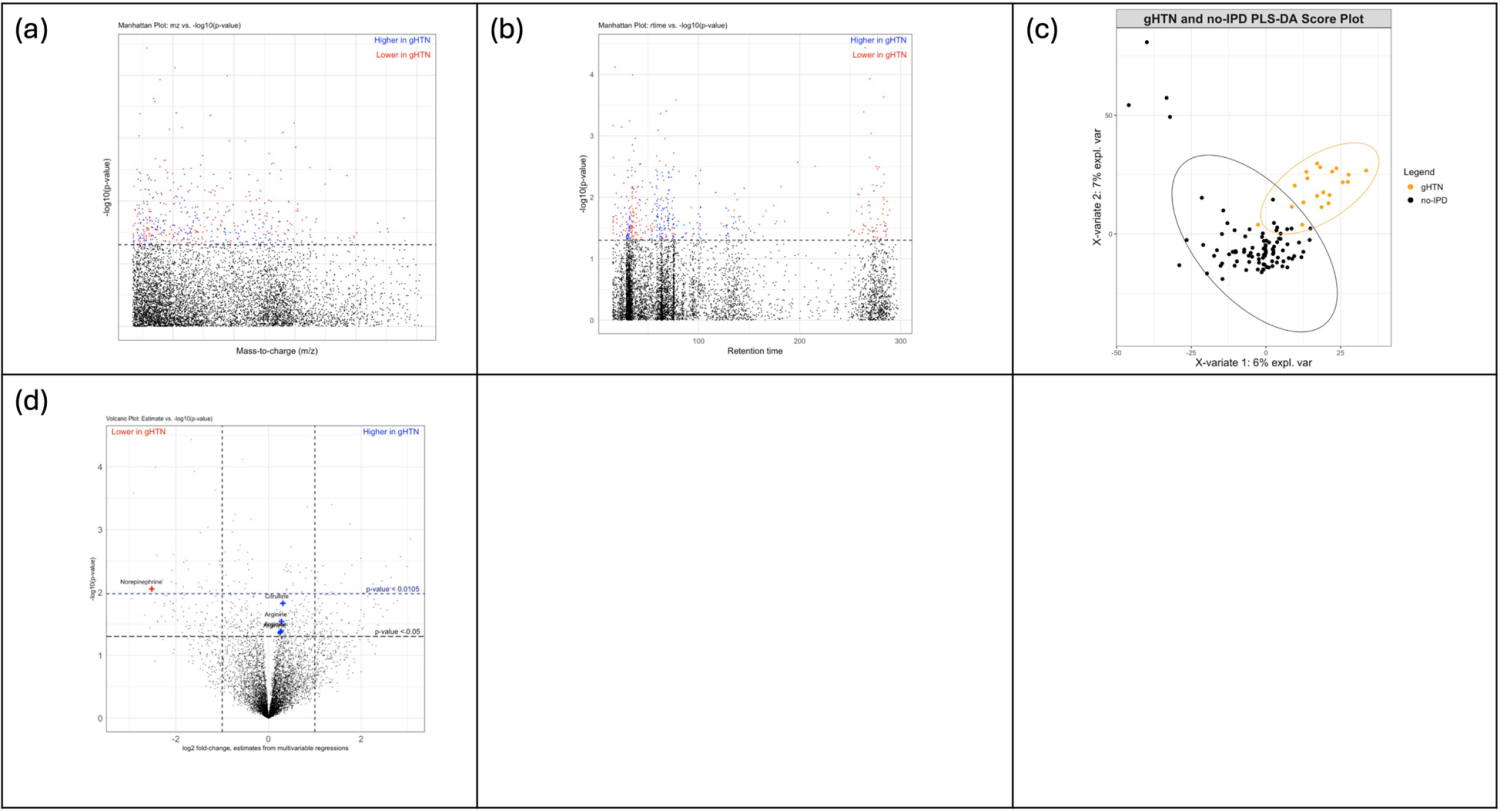
**a** Type 1 Manhattan Plot: This plot displays the -log10 p-values against mass-to-charge ratio (m/z). A total of 566 m/z features were found significant at a p-value of 0.05. Red dots indicate features less abundant in gHTN, while blue dots represent those more abundant in gHTN. The dashed line marks the significance threshold at p < 0.05. **b** Type 2 Manhattan Plot: -log10 p-values are plotted against rt. Most features exhibited a rt of less than 2 minutes. The dashed line again indicates the significance cut-off of p < 0.05. **c** PLS-DA Score Plot: Distinct clustering of gHTN (orange) and non-IPD (black) based on metabolic profiles. **d** Volcano Plot: Log2 fold changes from multivariable linear regressions are plotted on the x-axis against -log10(p-values) on the y-axis. The dashed line denotes the p-value threshold of 0.05 and 0.0105. Colored dots signify features with p < 0.05 and VIP > 1.5, highlighting metabolites considered for pathway enrichment analyses

**Fig. 3:**
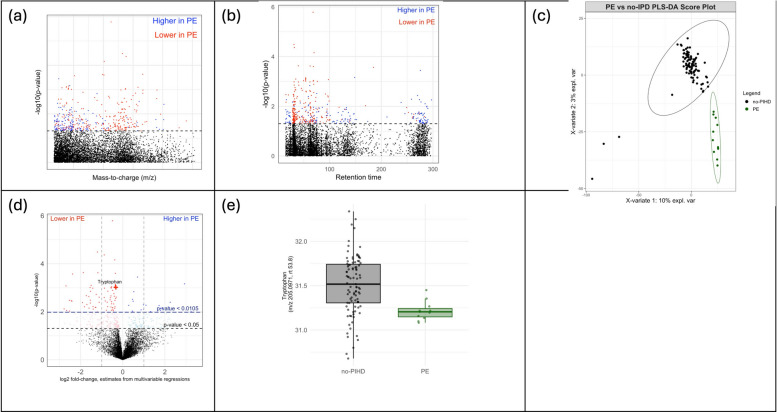
**a** Type 1 Manhattan plot: this plot displays the -log10 *p*-values against mass-to-charge ratio (*m/z*). A total of 415 m/z features were found significant at a *p*-value of 0.05. Red dots indicate features less abundant in preeclampsia (PE), while blue dots represent those more abundant in PE. The dashed line marks the significance threshold at *p* < 0.05. **b** Type 2 Manhattan Plot: -log10 *p*-values are plotted against rt. Most features exhibited a rt of less than 2 min. The dashed line again indicates the significance cut-off of *p* < 0.05. **c** PLS-DA Score Plot: there is no distinct clustering of PE (green) and non-IPD (black) based on metabolic profiles projected onto first component on x-axis and no PLS-DA model was built, discriminating PE vs non-IPD. **d** Volcano Plot: Log2 fold changes from multivariable linear regressions are plotted. The dashed line denotes the *p*-value threshold of 0.05 and  0.0105. Since no VIP was created colored dots signify features with *p* < 0.05 and *p* < 0.0105, m/z features with *p* < 0.0105 considered for pathway enrichment analyses. **e** Box and wisher plot: Tryptophan (m/z = 205.0971, rt = 53.8), negatively associated with PE as compared with non-IPD pregnancies, estimated effect size −0.33 log2 FC and 95%CI (−0.53, −0.14), *p* = 0.00097

We conducted MWAS comparing gHTN (20 samples) and non-IPD pregnancies (95 samples). A total of 566 features had a raw *p* value < 0.05 with a wide range of *m/z* and rt (Fig. 3(a) and (b)), of which 30 m*/z* features exhibited raw *p*-values < 0.0022; however, we were unable to annotate these features using our in-house library, and furthermore XMSannotator failed to annotate these features with high confidence. PLS-DA analysis identified 331 features with VIP scores > 1.5. The score plot shows distinct clustering of gHTN vs non-IPD pregnancies (Fig. 2(c)). A volcano plot illustrates the log_2_ FC for adjusted estimates from the multivariate linear regressions. We considered 204 features with both raw *p*-values < 0.05 and VIP scores > 1.5 (highlighted with colored dots) for pathway enrichment analyses (Fig. 2(d)). Statistically significant alterations were observed in the urea cycle/amino group metabolism and tyrosine metabolism pathways (*p*-value < 0.05) when comparing gHTN to non-IPD pregnancies (Table 2). Annotations of citrulline related metabolite (*m/z* = 159.0764, rt = 101.08, *p* = 0.013, and *m/z* = 77.1064, rt = 101, *p* = 0.06) and arginine (*m/z* = 175.119, rt = 129.4, *p* = 0.017) and its related metabolites (*m/z* = 129.1135, rt = 127.5, *p* = 0.0077 m*/z* = 88.0631, rt = 130.0, *p* = 0.01019; *m/z* = 158.0925, rt = 121.2, *p* = 0.0127; *m/z* = 176.1224, rt = 128.4, *p* = 0.034; and *m/z* = 88.5648, rt = 172.2, *p* = 0.018) in the urea cycle were further confirmed by correlating the distribution of these *m/z* features against confirmed arginine (Pearson-ρ > 0.93 and *p* < 0.001, for all five) and citrulline (Pearson-ρ 0.72 and 0.69, *p* < 0.001, for both) metabolites (Table 2, Supplementary Table 1 (Table 1S) and Supplementary Fig. 1(a) (Fig. S1a) and Fig. S1b). Additional features within this pathway included 4-aminobutanal (*m/z* = 88.0757, rt = 177.2, *p* = 0.0034), which was annotated using XMSannotator, and methionine (*m/z* = 150.0584, rt = 61.1, *p* = 0.0115), annotated using the in-house library, along with a related feature (*m/z* = 152.0549, rt = 60.4, *p* = 0.0129) that showed a strong correlation with methionine (ρ = 0.80, *p* < 0.001) (Fig. S1c)). Arginine, citrulline, and methionine were positively associated, while 4-aminobutanal was negatively associated with gHTN. In the tyrosine metabolism pathway, the annotation of 1,2-dehydrosalsolinol (*m/z* = 178.0862, rt = 58.6, *p* = 0.0021) and tyrosine (*m/z* = 182.0812, rt = 66, *p* = 0.0063) was further confirmed by XMS-annotator. The intensity of 1,2-dehydrosalsolinol was lower among the gHTN group, while tyrosine showed a positive association with gHTN (Table 1S). Pathway enrichment analyses also identified arginine and proline metabolism and aspartate and asparagine metabolism. These pathways shared several empirical features with the urea cycle and tyrosine metabolism, reflecting overlapping metabolite mapping rather than independent pathway-specific signals. Annotated metabolites were used to support pathway-level interpretation, and enrichment across multiple pathways primarily reflected repeated mapping of shared metabolites. Details of metabolite annotation and confirmation are provided in Table 1S**.**

**Table 2:** Summary of MWAS significant pathways and metabolite confirmation status

gHTN vs non-IPD							
Pathways*	Overlap size	Pathway size	*P* value	Overlap Empirical Compounds	N0 empirical confirmed	Anchor metabolites	Pathway supported
Urea cycle/amino group metabolism	11	49	5e-04	E1828,E1771,E1147,E2320,E106,E2569,E1461,E1469,E2203,E2290,E1905	5	4-Aminobutanal; L-Arginine; L-Citrulline; L-Methionine; Ornithine	TRUE
Arginine and Proline Metabolism	7	30	0.0037	E2290,E1147,E2320,E1461,E2569,E1771,E1905	4	4-Aminobutanal; L-Arginine; L-Citrulline; L-Methionine	TRUE
Aspartate and asparagine metabolism	10	60	0.00686	E18,E1771,E1147,E2467,E2320,E1963,E1461,E2203,E2290,E1905	5	4-Aminobutanal; 5-L-Glutamyl-L-alanine; L-Arginine; L-Citrulline; Ornithine	TRUE
Ubiquinone Biosynthesis	2	5	0.02576	E2660,E2003	NA	NA	NA
Alanine and Aspartate Metabolism	3	12	0.03076	E2320,E1461,E1905	NA	NA	NA
Tyrosine metabolism	10	77	0.03313	E1828,E489,E2660,E903,E106,E1125,E2693,E1311,E1469,E1213	3	1,2-dehydrosalsolinol; L-Tyrosine; Norepinephrine	TRUE
Limonene and pinene degradation	2	6	0.03812	E2404,E745	NA	NA	NA

PE vs non-IPD							
Pathways	Overlap size*	Pathway size	*P* value	Overlap Empirical Compounds	N0 empirical confirmed	Anchor metabolites	Pathway supported
Tryptophan metabolism	5	57	0.01118	E1033,E187,E1819,E1875,E1484	2	3-Methoxytyrosine; L-Tryptophan	TRUE
Caffeine metabolism	2	6	0.00546	E2037,E2415	NA	NA	NA
Pentose and Glucuronate Interconversions	2	6	0.00546	E1535,E2415	NA	NA	NA
Ascorbate (Vitamin C) and Aldarate Metabolism	2	13	0.02464	E1535,E2415	NA	NA	NA
Pentose phosphate pathway	2	19	0.04689	E1535,E2415	NA	NA	NA

PE vs gHTN							
Pathways	Overlap size*	Pathway size	*P* value	Overlap Empirical Compounds	N0 empirical confirmed	Anchor metabolites	Pathway supported
Tryptophan metabolism	13	57	0.00882	E115,E313,E1618,E2532,E1457,E29,E2569,E1153,E78,E1466,E760,E1372,E1046	2	2-Quinolinecarboxylic acid; D-Tryptophan	TRUE
Glycine, serine, alanine and threonine metabolism	11	46	0.01033	E2584,E1170,E1649,E1802,E915,E1439,E29,E2080,E1969,E1732,E2637	2	L-Arginine; L-Methionine	TRUE
Vitamin B3 (nicotinate and nicotinamide) metabolism	5	14	0.01197	E2584,E1432,E1372,E29,E322	2	Glutamine; L-Arginine	TRUE
Glycosphingolipid biosynthesis—neolactoseries	2	3	0.02344	E2224,E2161	NA	NA	NA
Keratan sulfate biosynthesis	2	3	0.02344	E2224,E2161	NA	NA	NA
O-Glycan biosynthesis	2	3	0.02344	E2224,E2161	NA	NA	NA
Proteoglycan biosynthesis	2	3	0.02344	E2224,E2161	NA	NA	NA
Glycosphingolipid biosynthesis—lactoseries	2	3	0.02344	E2224,E2161	NA	NA	NA
Blood Group Biosynthesis	2	3	0.02344	E2224,E2161	NA	NA	NA
Nitrogen metabolism	3	7	0.02445	E1432,E29,E322	NA	NA	NA
Alanine and Aspartate Metabolism	4	12	0.02729	E2584,E1432,E29,E322	2	3-Ureidoisobutyrate; L-Arginine	TRUE
Glutamate metabolism	4	12	0.02729	E29,E1432,E322,E1649	1	3-Ureidoisobutyrate	TRUE
Glycosphingolipid biosynthesis—ganglioseries	3	8	0.03492	E2224,E1693,E2161	NA	NA	NA
Glycosphingolipid biosynthesis—globoseries	3	8	0.03492	E2224,E1693,E2161	NA	NA	NA
Urea cycle/amino group metabolism	10	49	0.03539	E2584,E1144,E1439,E29,E85,E915,E1334,E2497,E2637,E1649	3	L-Arginine; L-Methionine; Norepinephrine	TRUE
Aminosugars metabolism	5	19	0.03856	E2224,E1432,E1693,E29,E322	2	3-Ureidoisobutyrate; UDP-N-acetyl-D-mannosamine	TRUE
Butanoate metabolism	6	25	0.03955	E1360,E2079,E29,E1170,E1649,E1981	1	N6-Acetyl-L-lysine	TRUE
Biopterin metabolism	3	9	0.04501	E2035,E1401,E2483	NA	NA	NA

*NA* not applicable

^*^Pathways were considered supported when at least four empirical compounds contributed to enrichment and at least one metabolite within the pathway was confirmed through annotation among overlapping empirical features

### Preeclampsia vs. non-IPD

**Fig. 4:**
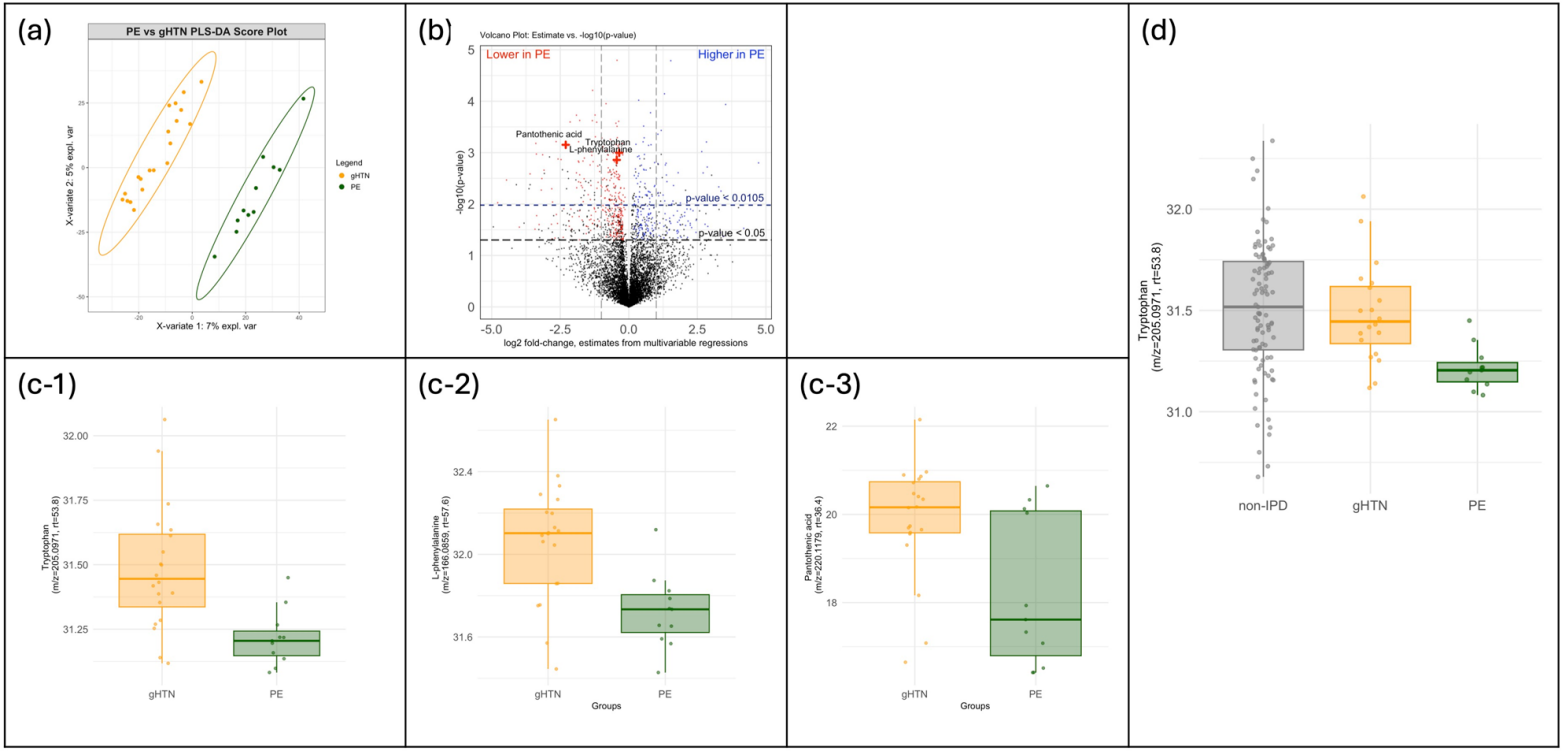
**a** PLS-DA score plot: distinct clustering of gHTN (orange) and PE (green) based on metabolic profiles. **b** Volcano Plot: Log2 fold changes from multivariable linear regressions are plotted on the x-axis against -log10(*p*-values) on the y-axis. The dashed line denotes the *p*-value threshold of 0.05 and 0.0105. Colored dots signify features with *p* < 0.05 and VIP > 1.5, highlighting metabolites considered for pathway enrichment analyses. **c** Box and whisker plots: for 3 significant verified features (from left to right) Tryptophan (*m/z* 205.0971; rt 53.8 s), L-phenylalanine (*m/z* 166.0859, rt 57.6 s) and Pantothenic acid (*m/z* = 220.1179, rt = 36.4), with *p*-value < 0.0022 for all. **d** Box and whisker plots: Tryptophan (*m/z* 205.0971; rt 53.8 s) across non-IPD, gHTN and PE pregnancies

Using multivariate linear regression, we identified 415 m*/z* features with raw *p*-values < 0.05 comparing PE cases (11 samples) and non-IPD pregnancies (95 samples) (see Fig. 4(a) and (b)). From these analyses, 32 m*/z* features met the criteria of an FDR-adjusted *p*-value threshold of 0.05 (raw *p*-value < 0.0022). Among these *m/z* features, tryptophan (m/z = 205.0971, rt = 53.8, *p* = 0.00097) was confirmed as an in-house library metabolite with an estimated effect size of −0.33 (95% CI: −0.53, −0.14). When performing PLS-DA, the first latent component of the model was not significant, precluding us from determining VIP scores (Fig. 3(c)). We performed pathway analyses on 91 features with raw *p*-value < 0.0105 and found the tryptophan metabolism pathway to be altered in early pregnancy in association with PE. From this pathway, tryptophan and four related metabolites (*m/z* = 188.0707, rt = 53.8, *p* = 0.0008; and *m/z* = 206.1005, rt = 53.7, *p* = 0.00072; *m/z* = 243.0528, rt = 54.9, *p* = 0.0026; and *m/z* = 159.0905, rt = 59.3, *p* = 0.0047) were negatively associated with PE. These features were annotated within the tryptophan pathway by Mummichog and showed strong correlations with in-house confirmed tryptophan Pearson ρ > 0.72 and *p* < 0.001, for all four) (Fig. S1e). 3-Methoxytyrosine (*m/z* = 212.0917, rt = 59.9, *p* = 0.009) was positively associated with PE (Table 1S).

### Preeclampsia vs. gestational hypertension

Multivariate linear regression analyses comparing PE cases (11 samples) versus gHTN cases (20 samples) identified 857 features with raw *p*-values < 0.05, of which 101 m*/z* features had *p*-values < 0.0022. Among these FDR significant features, tryptophan (*m/z* = 205.0971, rt = 53.8, *p* = 0.0010) and pantothenic acid (*m/z* = 220.1179, rt = 36.4, *p* = 0.00071), annotated using the in-house library, and L-phenylalanine (*m/z* = 166.0859, rt = 57.6, *p* = 0.0014), annotated at confidence level 3 using XMSannotator, had negative associations with PE and lower concentrations in PE. Tryptophan showed an estimated effect size of − 0.35 (95% CI − 0.54, − 0.16), pantothenic acid showed an estimated effect size of − 2.3 (95% CI − 3.51, − 1.09), and L-phenylalanine showed an estimated effect size of − 0.44 (95% CI − 0.68, − 0.19) (Fig. 4-c1 through c3). Subsequently, PLS-DA analysis identified 744 m*/z* features with VIP scores > 1.5. Pathway analysis encompassed 492 features meeting both criteria (*p* < 0.05 and VIP > 1.5), and alterations were again observed in the tryptophan metabolism pathway when comparing these two subtypes of PIHD (Fig. 4(c), Table 2). Tryptophan (mz = 205.0971, rt = 53.8) and its related metabolites (*m/z* = 188.0707, rt = 53.8; *m/z* = 206.1005, rt = 53.7; m/z = 243.0528, rt = 54.9; and m/z = 159.0905, rt = 59.3, Pearson-ρ > 0.72 and *p* < 0.001, for all four) within the tryptophan pathway, had lower concentration in PE than gHTN (Table 1S and Fig. S1e). In addition to the tryptophan pathway, the urea cycle/amino group metabolism pathway was also enriched in the comparison between PE and gHTN. Arginine (*m/z* = 175.119, rt = 129.4), its related metabolite (*m/z* = 129.1135, rt = 127.5), and methionine (*m/z* = 150.0584, rt = 61.1) with its related metabolites (*m/z* = 104.0528, rt = 60 and *m/z* = 152.0549, rt = 60.4, p, Pearson-ρ > 0.8 and *p* < 0.001, for both) (Fig. S1c) confirmed using the in-house library and served to support pathway-level enrichment and they showed positive association with PE. Except for tryptophan, individual metabolite-level associations did not remain statistically significant after false discovery rate correction and are therefore interpreted as suggestive. A summary of confirmed pathways by comparison group is presented in Table 2, while Table 1S provides expanded empirical compound-level annotation and feature-level confirmation details for MWAS-supported pathways**.**

### Tryptophan levels across groups: non-IPD, gHTN, and PE

We found a statistically significant linear trend in tryptophan (*m/z* = 205.0971, rt = 53.8) across the groups with non-IPD, gHTN, and PE. In multivariable linear regression modeling complication status as an ordinal variable, each one-category increase in disease severity was associated with a − 0.12 decrease in log2 tryptophan concentration (β = − 0.116, SE = 0.043, *p* = 0.008), indicating a dose–response relationship. Median (IQR) log2 concentrations of tryptophan were [31.5 (31.3–31.7)], [31.4 (31.3–31.6)], and [31.2 (31.1–31.2)] for the non-IPD, gHTN, and PE groups, respectively (Fig. 4(d)). These differences correspond to an approximate 7% decrease in median tryptophan concentrations between non-IPD and gHTN, with a further 13% decrease between gHTN and PE on the linear scale.

In adjusted pairwise comparisons, PE was associated with lower tryptophan concentrations compared to non-IPD (β = −0.33, 95% CI: −0.53, −0.14, *p* = 0.00097), and compared to gHTN (β = −0.35, 95% CI: −0.54, −0.16, *p* = 0.001), consistent with the observed monotonic trend.

## Discussion

Our study identified distinct metabolic profiles in maternal serum associated with gHTN (14 mothers; 20 samples) and PE (9 mothers; 11 samples) compared to non-IPD (74 mothers; 95 samples) pregnancies early in gestation (10–20 weeks). Notably, while gHTN and PE share elevated BP as a common clinical feature, our findings identified a tryptophan-related pathway associated with both outcomes, alongside distinct metabolic signatures that differentiated PE from gHTN. We observed a potential dose-response in the tryptophan metabolism pathway, where tryptophan intensity was lowest in PE, while gHTN values fell between PE and non-IPD pregnancies. These results aligned mainly with past findings regarding the tryptophan metabolism pathway, and may represent varying severities along the same disease continuum [5]. However, we observed greater variation in the metabolic profiles when comparing PE to gHTN. This suggests that the two conditions may exhibit distinct metabolic patterns, which could reflect different underlying mechanisms or a metabolic response related to progression from gHTN to PE in pregnant women. Nonetheless, further investigation with a larger sample size in a diverse population is required to support these initial results.

Beyond tryptophan-related alterations, we also observed enrichment of the urea cycle metabolism pathway when comparing gHTN to non-IPD pregnancies, with positive associations between arginine and citrulline from urea cycle metabolism before 20 weeks of pregnancy, suggesting a role for these amino acids in hypertensive conditions [25]. Both arginine and citrulline are crucial intermediates in the urea cycle and are involved in the regulation of BP through vascular endothelial nitric oxide (NO) synthesis. Arginine is a direct precursor of NO, a potent vasodilator that mediates relaxation of vascular smooth muscle, thereby reducing resistance and improving blood flow [26, 27]. Citrulline, on the other hand, is involved in mediating the recycling of arginine, helping sustain NO-related signaling [28, 29]. This connection between the urea cycle metabolites and NO synthesis provides a mechanistic link to the regulation of vascular tone and BP, which has been implicated in hypertensive disorders such as gHTN, as reduced NO availability has been associated with endothelial dysfunction and hypertension [30–32]. Studies have shown that supplementation with L-arginine and L-citrulline can improve NO availability, enhance uterine-placental circulation, and potentially lower BP in pregnant women [33–35]. However, elevated circulating levels of arginine and citrulline do not necessarily indicate increased NO production. Accumulation of these intermediates may instead reflect altered metabolic flux, impaired enzymatic conversion, or reduced downstream utilization within the urea cycle or NO synthesis pathways. Thus, higher measured concentrations may coexist with reduced functional NO bioavailability, potentially reflecting dysregulated nitrogen handling or endothelial signaling rather than enhanced vasodilatory capacity. Consistent with this biology, altered urea cycle metabolites may contribute to endothelial dysfunction through reduced NO bioavailability, promoting vasoconstriction, oxidative stress, and heightened sensitivity to circulating vasopressors, which may contribute to the development of gHTN or PE [36, 37]. Although urea cycle amino group metabolism was enriched in both gHTN versus non-IPD and PE versus gHTN comparisons, the direction of individual metabolites differed between contrasts. Arginine and methionine were positively associated with gHTN relative to non-IPD, whereas both metabolites were negatively associated with PE when compared with gHTN. This pattern may suggest that enrichment of the same metabolic pathway reflects perturbation of a shared nitrogen handling network across hypertensive phenotypes rather than a simple monotonic dose-response. However, given the modest sample size and the exploratory nature of pathway enrichment analyses, these observations should be interpreted as hypothesis-generating rather than confirmatory. Overall, the observed pattern may indicate a metabolic divergence of PE from gHTN within the same pathway, which will require confirmation in larger and independent cohorts.

Tryptophan metabolism early in pregnancy may precede the development of PE. This is particularly noteworthy as tryptophan metabolism has been implicated in immune regulation and endothelial function, both of which play a critical role in the etiology of PE. Tryptophan is essential for increased protein synthesis to support placental and fetal growth and for suppressing immune responses to achieve maternal–fetal immune tolerance and prevent fetal rejection [38]. In our study, tryptophan and its related metabolites were predominantly negatively associated with PE, suggesting a potential depletion or increased utilization of these metabolites in pregnancies that later develop PE. Conversely, 3-Methoxytyrosine (3-MT), a metabolite within the tryptophan pathway, was positively associated with PE. 3-MT is a byproduct of catecholamine metabolism. Elevated levels of catecholamines have been associated with stress responses and hypertension, which are relevant in pregnancy-related conditions like PE and gHTN [39].

We also saw quantitative differences related to the tryptophan pathway comparing PE to gHTN that further underscored the importance of tryptophan metabolism in early gestation before PE develops [40, 41]. We observed a statistically significant dose–response relationship in tryptophan concentrations across increasing disease severity, with a 7% reduction from non-IPD to gHTN and an additional 13% decrease from gHTN to PE. Building on the pattern described above, these quantitative results further characterize a graded alteration in tryptophan-related metabolism across increasing clinical severity. Thus, while gHTN and PE share this metabolic feature, the depletion or altered utilization of tryptophan is more pronounced in PE, highlighting its potential role in the pathophysiology of more severe hypertensive disorders in pregnancy. To our knowledge, no study compares circulating tryptophan concentrations across healthy, gHTN, and PE women explicitly; however, past literature indicates a general negative association between tryptophan and BP [42].

1,2-Dehydrosalsolinol was significantly lower in gHTN pregnancies compared to non-IPD (*p* = 0.0021) ones, being identified within the tryptophan metabolism pathway during enrichment analysis. This metabolite is a byproduct of dopamine metabolism and can influence monoamine oxidase activity, which is responsible for the breakdown of several neurotransmitters, including dopamine and tyramine. While we do not have direct measurements for dopamine or tyramine available, these related metabolites are critical for understanding the broader context of monoamine metabolism in gHTN. To further investigate this finding, we explored additional metabolites related to this pathway, including tryptophan and 5-hydroxy-L-tryptophan, which are precursors to serotonin. We found no significant differences for tryptophan (−0.03 [−0.2, 0.15], *p* = 0.8), kynurenine (−0.07 [−0.32, 0.18], *p* = 0.6), and cortisol (−0.43 [−1.34, 0.47], *p* = 0.31); however, 5-hydroxy-L-tryptophan levels were significantly lower in gHTN compared to non-IPD (−1.14 [−2.01, −0.26], *p* = 0.011). The significant findings for both 1,2-dehydrosalsolinol (−0.65 [−1.06, −0.24], *p* = 0.002) and 5-hydroxy-L-tryptophan strengthen the hypothesis that the disruption in the tryptophan pathway, as well as monoamine metabolism, is not random but may be a consistent feature of gHTN. This relationship may be relevant to BP regulation, as intrarenal synthesis of serotonin from 5-hydroxy-L-tryptophan has been shown to influence renal hemodynamics and perfusion pressure [43]. Although our study did not measure serotonin directly, reduced availability of its precursors could plausibly contribute to altered serotonergic activity that has been implicated in hypertensive disorders [44].

The reduced levels of tryptophan and its related metabolites, including 2-quinolinecarboxylic acid, in pregnancies that later developed PE are consistent with previous research, indicating that changes in tryptophan metabolism throughout pregnancy may correlate with PE's distinctive immunological profile that plays a critical role in the progression of this condition [45]. The decreased concentrations of 2-quinolinecarboxylic acid in maternal serum during early gestation, well before clinical diagnosis, suggest that these metabolic changes occur early in pregnancy. This observation is consistent with reports of lower concentrations of this metabolite in placental tissue from PE pregnancies compared to uncomplicated pregnancies [46]. Although not statistically significant, our findings of lower kynurenine amounts in maternal serum during early gestation in pregnancies that subsequently developed PE or gHTN compared to non-IPD, and lower concentrations in PE compared to gHTN, align with prior studies [47]. Together, these results suggest that the alterations or depletion of metabolites in the kynurenine pathway during early gestation may serve as a risk factor for gHTN and PE. While the exact relationship still requires further exploration, it has been proposed that tryptophan (and downstream metabolites in the kynurenine pathway) is essential for pregnancy adaptation, due to its role in early placentation, vasodilatation of uterine vessels, and immune adaptation throughout pregnancy [42, 48]. In a mouse model, impaired placental perfusion and ischemia were associated with an inability to upregulate the L-tryptophan-derived L-kynurenine pathway, linking lower kynurenine concentrations to placental dysfunction [47]. However, a separate human study in an African American cohort reported a contrary finding of higher kynurenine concentrations in PE compared to gHTN, based on maternal serum samples collected during the first trimester of pregnancy, a finding that could not be directly replicated in our study because our cohort did not include an adequate sample size of African American women with PE [5]. These prior studies collectively suggest that disruption of the tryptophan pathway may exacerbate inflammatory and oxidative stress responses, contributing to the pathophysiology of PE.

Lastly, we found evidence of altered glycine and alanine metabolic pathways when comparing the PE and gHTN groups, which aligns with recent studies exploring signals associated with PE expressing more severe features (e.g., cardiovascular conditions) [48, 49]. This may also imply a difference in the branched-chain amino acid profile in women with PE, though further investigation is needed to confirm its pathophysiological involvement [49, 50].

We identified two additional metabolites, L-phenylalanine, and pantothenic acid, as being lower in PE than gHTN. In a study where maternal serum was collected between 11–14 weeks of gestation, phenylalanine was reported to be elevated in maternal serum in pregnancies that subsequently developed PE compared to normal pregnancies [51]; however, another study noted lower phenylalanine amounts in all stages of pregnancy, including the first trimester, with pregnancies that resulted in gHTN compared to those who did not [11, 52]. These discrepancies in concentrations may be due to differences in study populations or in the sensitivity of detection methods used across studies. Pantothenic acid, a precursor of coenzyme A involved in central energy and amino acid metabolism, was also lower in PE compared with gHTN. Although pantothenate-specific pathways were not enriched in our analysis, altered concentrations of pantothenic acid may reflect broader disruptions in amino acid and energy-related metabolic processes, which are consistent with the pathways identified in the PE versus gHTN comparison [53, 54]. Unique aspects of our study, such as collecting samples before 20 weeks of gestation, prior to the clinical diagnosis of PE or gHTN, and adjusting for confounders like maternal age, race, fetal sex, parity, and early-pregnancy BMI, may explain the differences observed compared to studies with alternate sampling times or adjustment strategies.

To assess the robustness of our findings, we repeated the pathway enrichment analyses using models with robust standard errors to account for the small number of mothers contributing more than one sample. For gHTN vs. non-IPD, the same top pathways, including Urea cycle/amino group metabolism, remained enriched. In both PE vs. non-IPD and PE vs. gHTN comparisons, tryptophan metabolism remained the primary altered pathway. These consistent pathway level results suggest that the observed metabolic alterations were not driven by model specification and were robust across analytic approaches. As an additional sensitivity analysis, we repeated the enrichment analyses after excluding pregnancies with early low-dose aspirin exposure, as some mothers received prophylactic low-dose aspirin to reduce the risk of hypertensive disorders of pregnancy, which may influence inflammatory and metabolic pathways [55]. Pathway level findings remained stable across sensitivity analyses. After excluding pregnancies with early low-dose aspirin exposure, the gHTN vs. non-IPD comparison continued to show enrichment of urea cycle and amino group metabolism as the primary altered pathway. In both PE vs. non-IPD and PE vs. gHTN comparisons, tryptophan metabolism remained the dominant enriched pathway. Similar patterns were observed in models using robust standard errors, with consistent pathway ranking and overlapping MWAS features contributing to enrichment. Overall, these sensitivity analyses supported the stability of the pathway enrichment results and suggested that the observed metabolic signatures were not driven by aspirin exposure or modeling assumptions.

The strengths of our study include the use of an advanced metabolomics platform that enabled the robust identification of metabolites across a broad spectrum of *m/z* and rt. Moreover, our cohort consisted of racially diverse mothers, some with high-risk pregnancies, and was prospective in terms of serum sampling and outcomes assessment, and we considered a range of possible confounders. Our study is limited by its relatively small sample size, given the prospective nature of the study and a one-site clinical trial. Additionally, the untargeted metabolomics approach presented challenges in metabolite annotation, as some features could not be confidently identified due to the lack of corresponding reference standards. Furthermore, our study focused exclusively on metabolomics data, and it is important to acknowledge that lipidomics have also shown alterations related to pregnancy complications like PE. While there were no differences in gestational diabetes mellitus (GDM) and BMI between the groups, and our models were adjusted for BMI, exclusion of participants with BMI > 30 or GDM was not feasible given sample size constraints. Therefore, our findings should be considered preliminary and need to be validated in larger cohorts preferably with both metabolomic and lipidomic data.

## Conclusion

In conclusion, our study provides valuable evidence that distinct metabolic alterations are associated with gHTN, PE, and non-IPD. These preliminary findings may pave the way for the eventual development of metabolomic biomarkers that can define endophenotypes prospectively and possibly even enable screening and closer vigilance and monitoring of at-risk pregnancies during early gestation. Ultimately, better stratification of subjects in clinical studies may yield success in detecting appropriate and targeted interventions for gHTN separate from PE.

## Supplementary Information

Supplementary Material 1.

Supplementary Material 2.

## Data Availability

Data required to reproduce the findings herein will be shared via the Human Health Exposure Analysis Resource (HHEAR, formerly CHEAR) platform maintained by the National Institute of Environmental Health Sciences (NIEHS) contractors.

## Abbreviations

3-DT: 3-Dimensional trophoblast assay
ANOVA: Analysis of Variance
BMI: Body mass index
BP: Blood pressure
CHEAR: Children’s Health Exposure Assessment Resource
FC: Fold change
FDR: False discovery rate
FGR: Fetal growth restriction
gHTN: Gestational hypertension
GDM: Gestational diabetes mellitus
HDP: Hypertensive disorders of pregnancy
HRM: High-resolution metabolomics
IQR: Interquartile ranges
IPD: Ischemic placental disease
LC-HRMS: Liquid chromatography coupled with high-resolution mass spectrometry
m/z: Mass-to-charge ratio
MRI: Magnetic resonance imaging
MWAS: Metabolome-wide association study
NIEHS: National Institute of Environmental Health Sciences
NO: Nitric oxide
PARENTs: Placental Assessment in Response to Environmental Pollution study
PE: Preeclampsia
PIHD: Pregnancy-induced hypertensive disorder
PLS-DA: Partial least squares–discriminant analysis
rt: Retention time
SGA: Small‑for‑gestational‑age
UCLA: University of California, Los Angeles
VIP: Variable importance for projection
