# Supplementary material for: Serum metabolomics in early pregnancy differentiates between gestational hypertension and preeclampsia
Source: BMC Pregnancy Childbirth. 2026 Apr 29;26:649. doi: 10.1186/s12884-026-09168-0 (PMC13270631; doi:10.1186/s12884-026-09168-0)
Supplement: Supplementary file 2 — Supplementary Material 2. [file 12884_2026_9168_MOESM2_ESM.pdf]

supplementary figures

Figure S1. Confirmation of Empirical Metabolites Relative to In House Confirmed.

a) Citrulline Related Empirical Metabolites Relative to In House Confirmed Citrulline

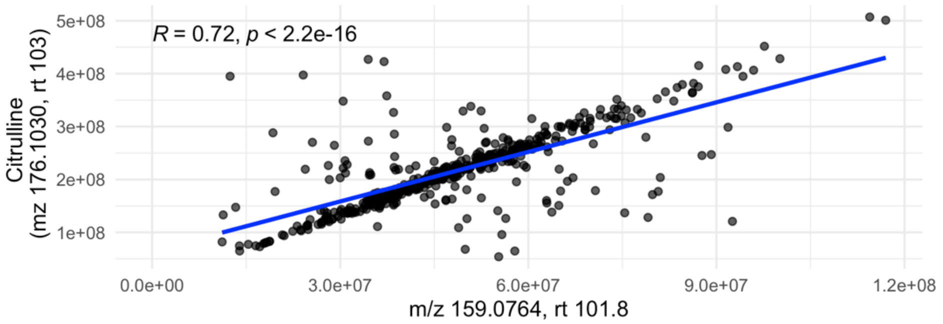

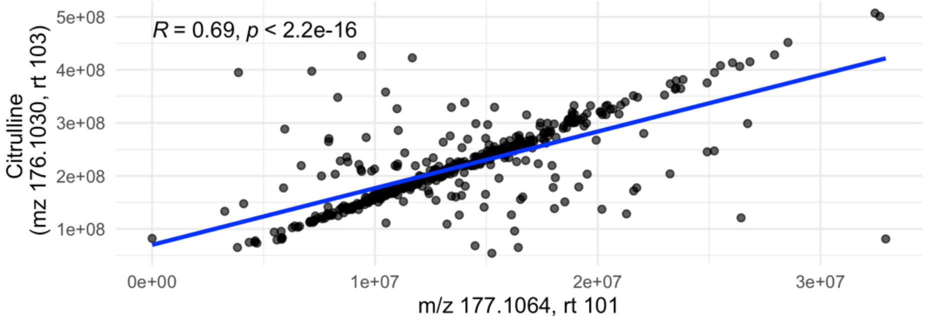

b) Arginine Related Empirical Metabolites Relative to In House Confirmed Arginine

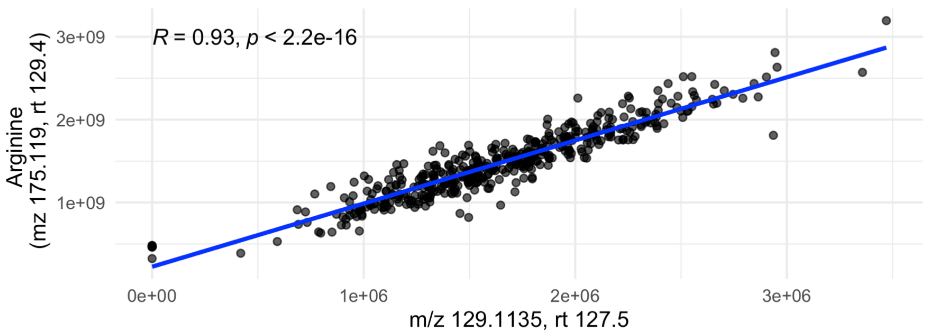
R = 0.93, p < 2.2e-16
Arginine
(mz 175.119, rt 129.4)
3e+09
2e+09
1e+09
0e+00
1e+06
2e+06
3e+06
m/z 129.1135, rt 127.5

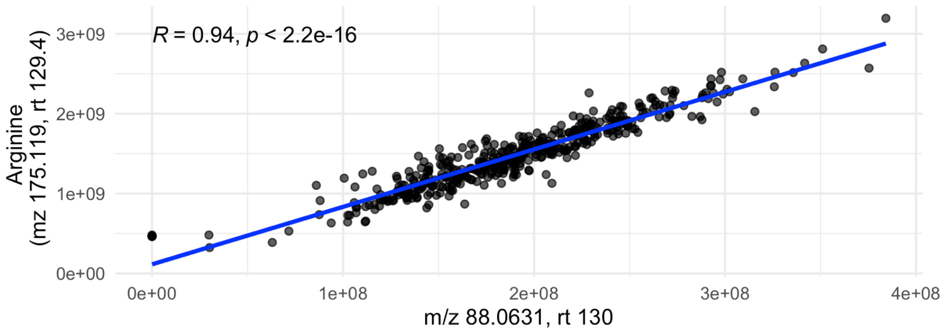
R = 0.94, p < 2.2e-16
Arginine
(mz 175.119, rt 129.4)
3e+09
2e+09
1e+09
0e+00
0e+00
1e+08
2e+08
3e+08
4e+08
m/z 88.0631, rt 130

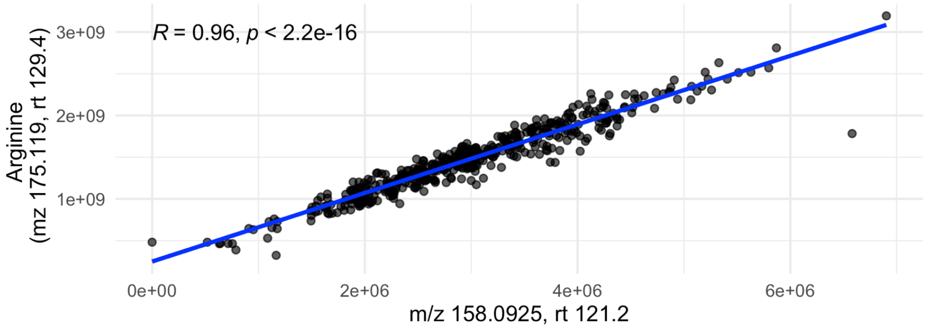
R = 0.96, p < 2.2e-16
Arginine
(mz 175.119, rt 129.4)
3e+09
2e+09
1e+09
0e+00
2e+06
4e+06
6e+06
m/z 158.0925, rt 121.2

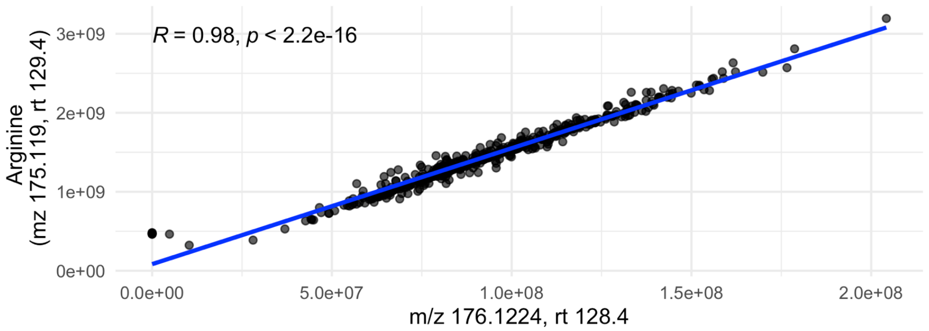
R = 0.98, p < 2.2e-16
Arginine
(mz 175.119, rt 129.4)
3e+09
2e+09
1e+09
0e+00
0.0e+00
5.0e+07
1.0e+08
1.5e+08
2.0e+08
m/z 176.1224, rt 128.4

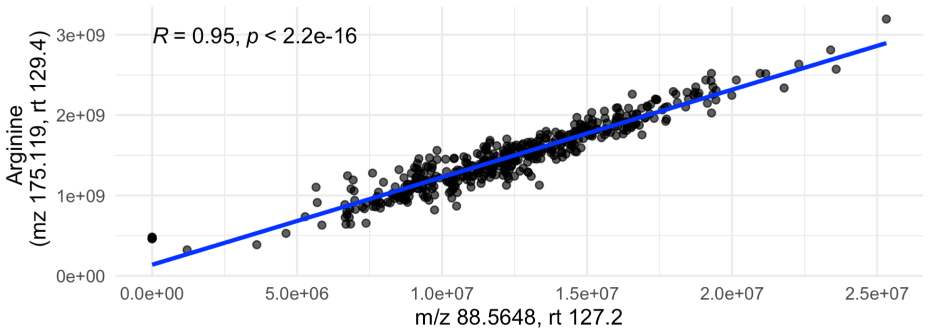
R = 0.95, p < 2.2e-16
Arginine
(mz 175.119, rt 129.4)
3e+09
2e+09
1e+09
0e+00
0.0e+00
5.0e+06
1.0e+07
1.5e+07
2.0e+07
2.5e+07
m/z 88.5648, rt 127.2

c) Methionine Related Empirical Metabolites Relative to In House Confirmed Methionine

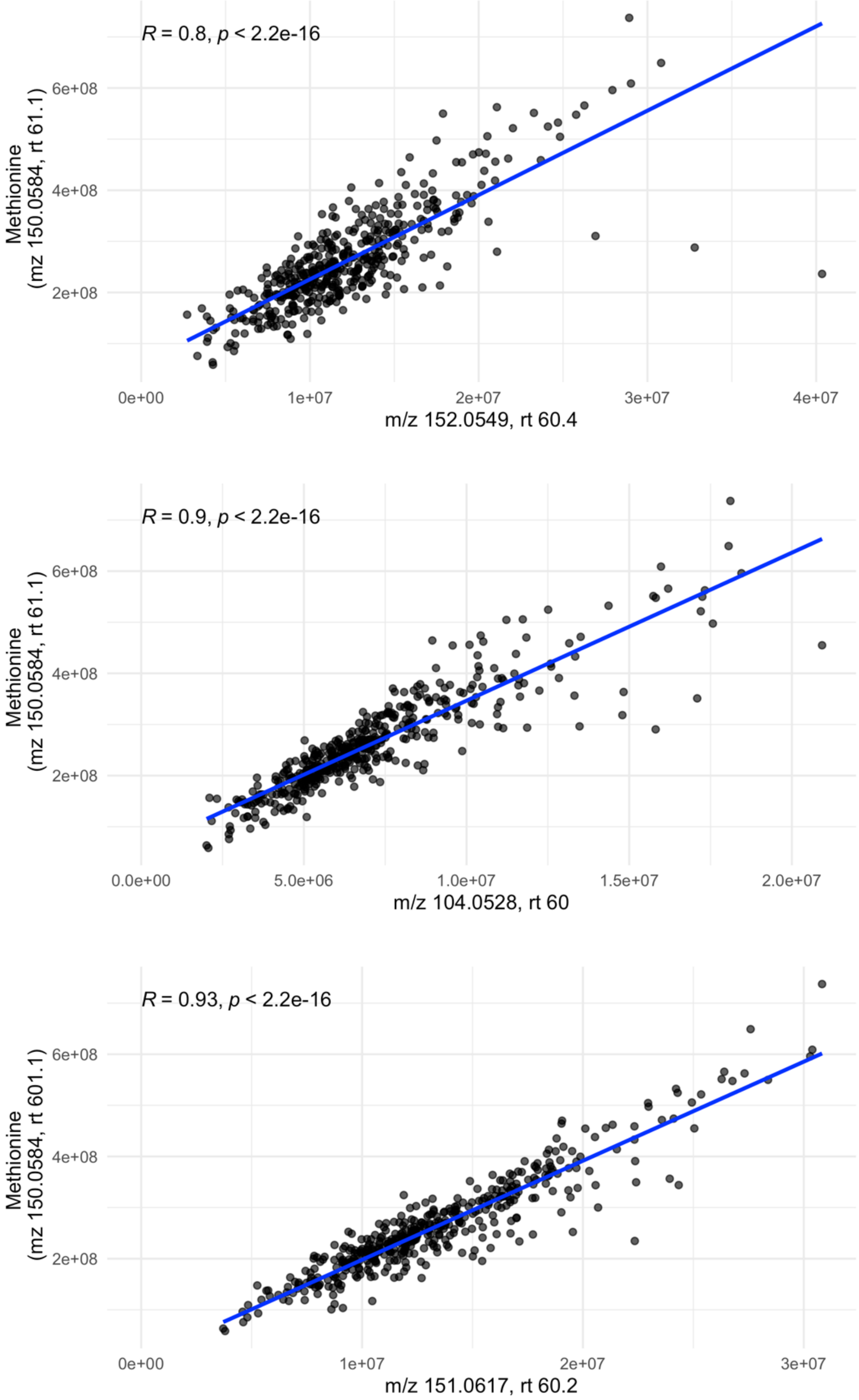

d) Ornithine Related Empirical Metabolites Relative to In House Confirmed Ornithine

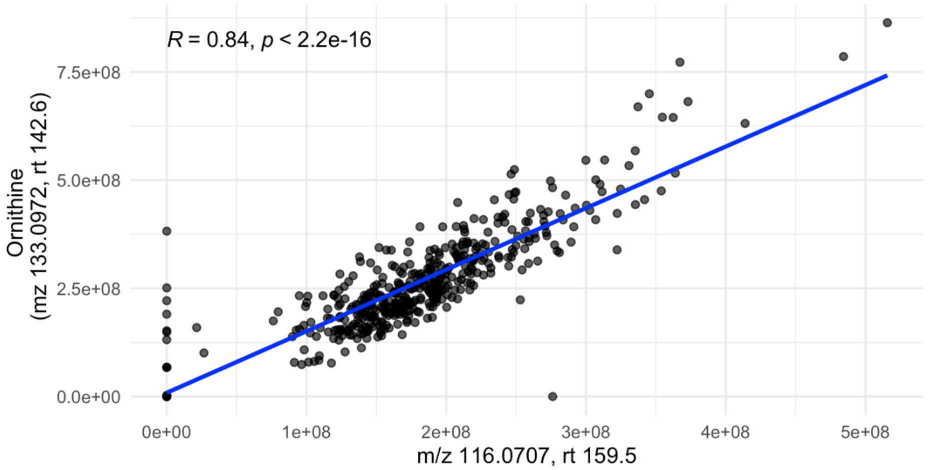

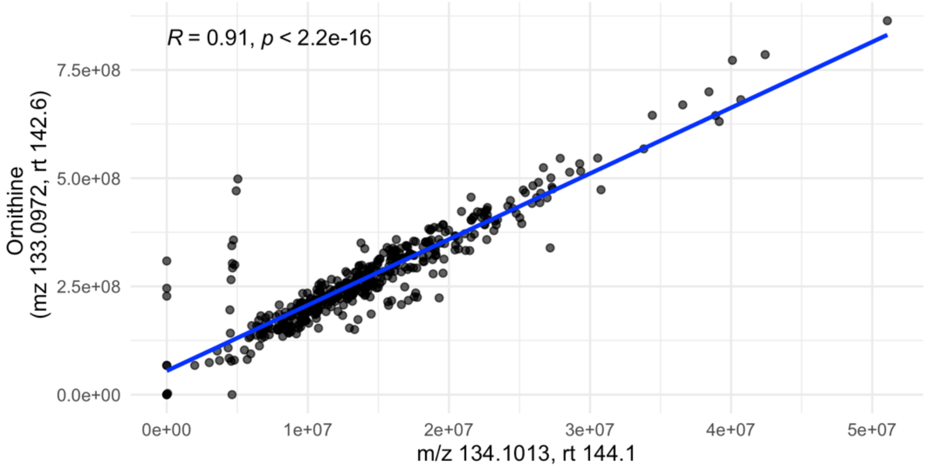

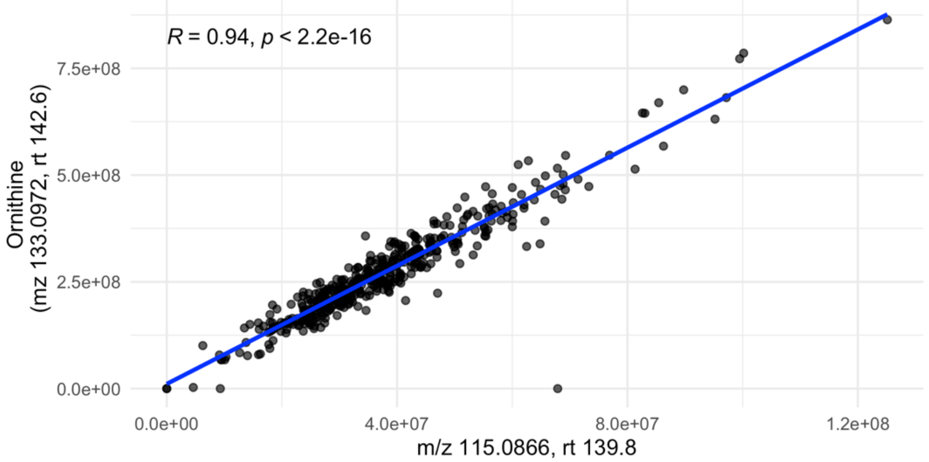

e) Tryptophan Related Empirical Metabolites Relative to In House Confirmed Tryptophan

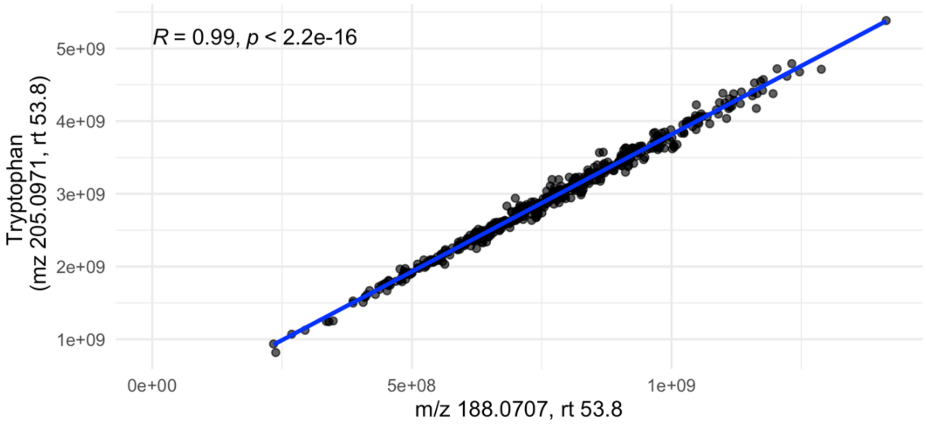

R = 0.99, p < 2.2e-16
Tryptophan (mz 205.0971, rt 53.8)
1e+09
2e+09
3e+09
4e+09
5e+09
0e+00
5e+08
1e+09
m/z 188.0707, rt 53.8

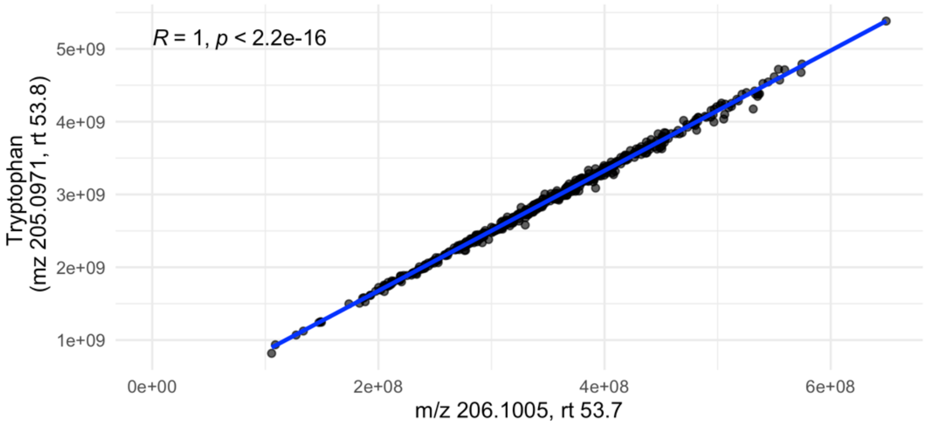

R = 1, p < 2.2e-16
Tryptophan (mz 205.0971, rt 53.8)
1e+09
2e+09
3e+09
4e+09
5e+09
0e+00
2e+08
4e+08
6e+08
m/z 206.1005, rt 53.7

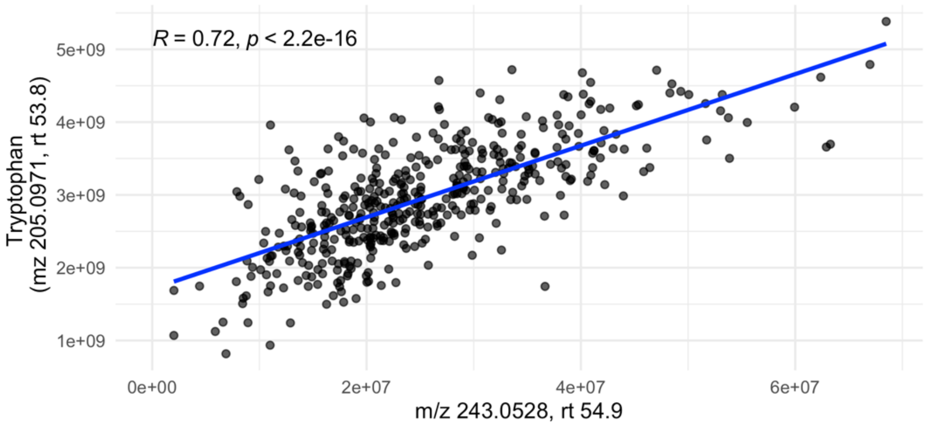

R = 0.72, p < 2.2e-16
Tryptophan (mz 205.0971, rt 53.8)
1e+09
2e+09
3e+09
4e+09
5e+09
0e+00
2e+07
4e+07
6e+07
m/z 243.0528, rt 54.9

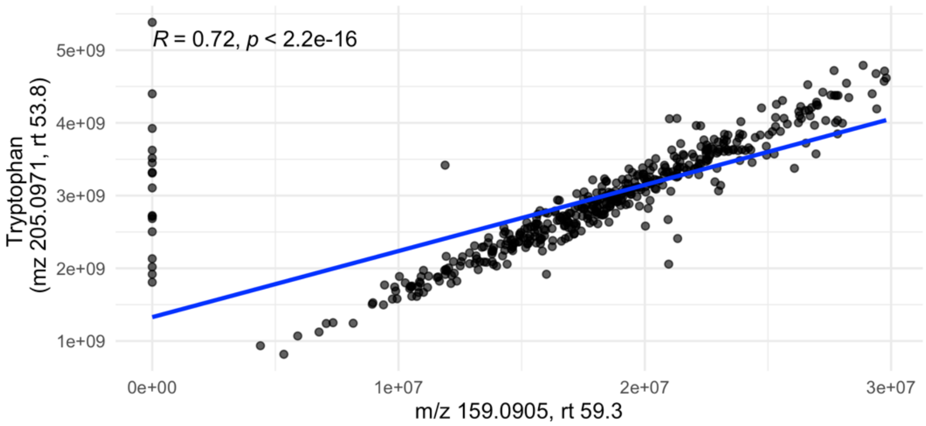

R = 0.72, p < 2.2e-16
Tryptophan (mz 205.0971, rt 53.8)
1e+09
2e+09
3e+09
4e+09
5e+09
0e+00
1e+07
2e+07
3e+07
m/z 159.0905, rt 59.3
